# ANCA-negative eosinophilic granulomatosis with polyangiitis in a girl misdiagnosed as asthma and pulmonary tuberculosis

**DOI:** 10.1186/s12969-023-00847-2

**Published:** 2023-06-21

**Authors:** Fang Yang, Jiwei Zhao, Jieying Wang, Jiangrong Liao, Jinlin Liu, Yanxia Chen

**Affiliations:** 1 Department of Respiratory Medicine, Guizhou Aerospace Hospital, Zunyi, China; 2 Department of Laboratory Medicine, Nanjing Lishui District Hospital of Traditional Chinese Medicine, Nanjing, China; 3 Department of Laboratory Medicine, The Second Affiliated Hospital of Hainan Medical University, Haikou, China; 4 Department of Clinical Laboratory, South China Hospital, Medical School, Shenzhen University, Shenzhen, China; 5 Department of Rheumatology and Immunology, South China Hospital, Medical School, Shenzhen University, 1 Fuxin Road, Shenzhen, 518111 China

**Keywords:** Eosinophilic granulomatosis with polyangiitis; Asthma; Pulmonary tuberculosis

## To the Editor:

Eosinophilic granulomatosis with polyangiitis (EGPA) is a rare form of pediatric systemic vasculitis. Owing to the atypical clinical manifestations and limited recognition of this disorder, delayed or missed diagnosis of pediatric EGPA is common [1, 2]. Herein, we describe the case of a 19-year-old girl with EGPA and recurrent wheezing and breathlessness, who was misdiagnosed with asthma and pulmonary tuberculosis for 3 years. Her pulmonary symptoms were not well controlled, although she had received inhaled glucocorticosteroids for more than two years and anti-tuberculosis drugs for 3 weeks. A lung biopsy was performed and eosinophilic pneumonia was diagnosed. When bilateral purpura in the lower limbs appeared during hospitalization, EGPA was suspected, although the anti-neutrophil cytoplasmic antibody (ANCA) results were negative, and EGPA was finally confirmed by skin biopsy. Glucocorticoid therapy was initiated and was found to be effective. Thus, this case highlights the fact that pediatricians should be aware of the possibility of EGPA in children with long-standing asthma.

**Fig. 1:**
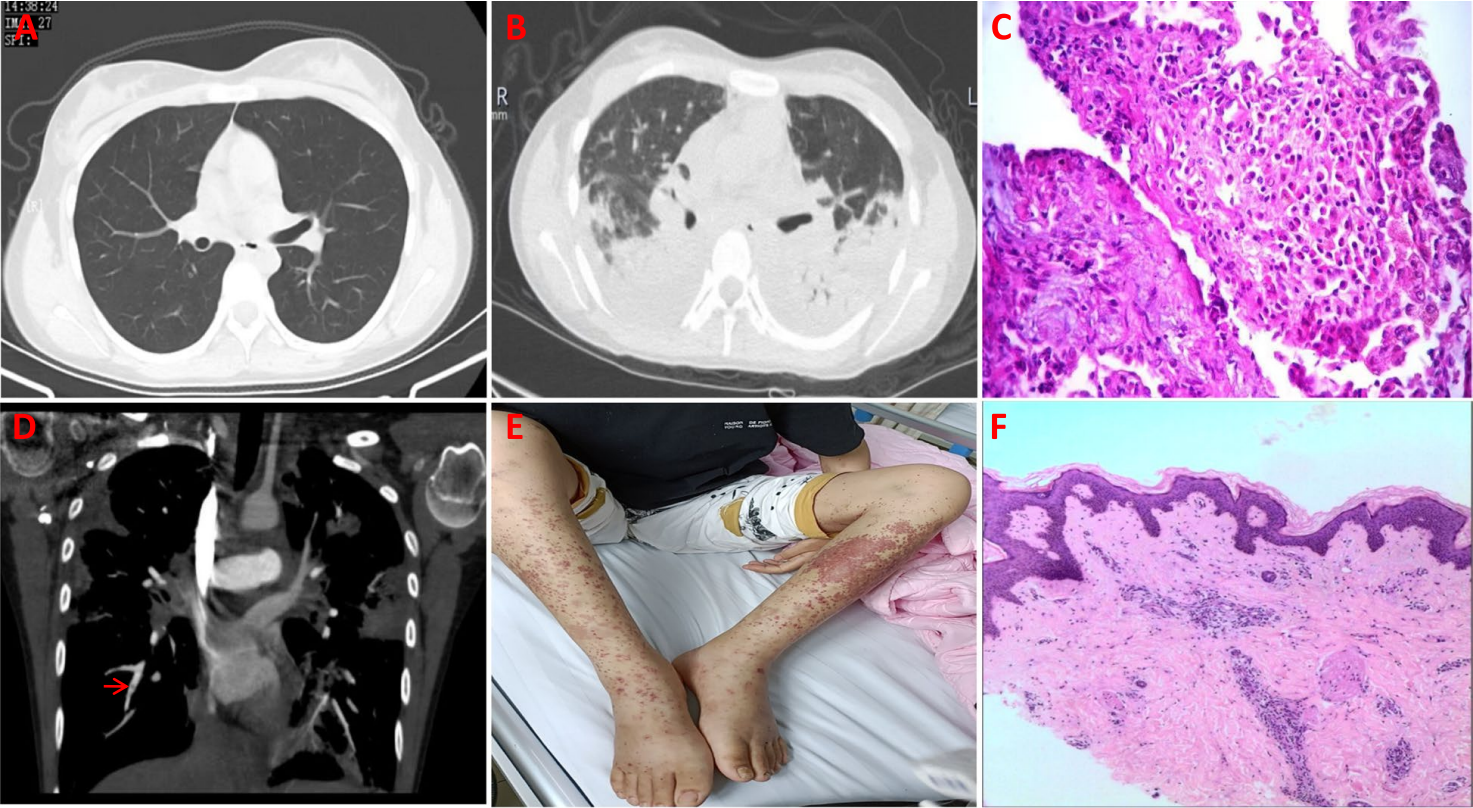
A delayed diagnosis of a patient with ANCA-negative eosinophilic granulomatosis with polyangiitis. **A** Chest CT scan showed a normal lung. **B** Chest CT showed bilateral lung patchy exudation. **C** Lung biopsy revealed eosinophilic and granulomatous inflammation (magnification, 200 × , HE staining). **D** Lung artery CT revealed mild embolism (red arrow). **E** Skin lesion presenting as purpura in bilateral lower limbs. **F** Vasculitis was found on skin pathology (magnification, 40 × , HE staining)

In August 2020, a 16-year-old girl was admitted to a local clinic with recurrent wheezing and breathlessness. Examination revealed a normal lung computed tomography (CT) image (Fig. 1A), a high-level nitric oxide breath test, and hypereosinophilia. She was diagnosed with asthma and received inhaled glucocorticosteroids, but experienced episodic shortness of breath for three years.

In February 2023, this patient was admitted to two other hospitals, and was clinically diagnosed with pulmonary tuberculosis based on the clinical symptoms, lung CT image, and positive interferon-γ release assay, but without *Mycobacterium tuberculosis* or other microorganism isolation in blood or sputum. She had started antibiotic treatment (isoniazid, rifapentine, ethambutol, and pyrazinamide) for 3 weeks, but bilateral purpura in her lower limbs appeared after antibiotic drug use. The side effects of the antibiotics were considered, and these drugs were discontinued.

In March 2023, the same patient was admitted to our hospital with recurrent wheezing and breathlessness. Laboratory studies revealed prominent peripheral eosinophilia with a value of 2.13 × 10^9^/L (0.02–0.6 × 10^9^/L). CT chest showed bilateral lung patchy exudation (Fig. 1B). A lung biopsy was performed and pathological analysis revealed eosinophilic and granulomatous inflammation(Fig. 1C). Therefore, eosinophilic pneumonia was diagnosed. She was administered prednisone (up to 25 mg daily), which significantly relieved her respiratory symptoms. Interestingly, D-Dimer was significantly high with the value of 3.64ug/mL (0–0.55ug/mL) in this girl. Lung artery CT revealed a mild embolism of the lung artery (Fig. 1D). Furthermore, purpura in both lower limbs reappeared during prednisone administration (Fig. 1E). A skin biopsy was performed, and vasculitis was found on skin pathology (Fig. 1F). However, an ANCA test, which is a characteristic marker of ANCA-associated vasculitis, was negative. Finally, given her history of asthma, eosinophilia, eosinophilic pneumonia, and vasculitis as confirmed by skin biopsy, a diagnosis of ANCA-negative EGPA was confirmed.

EGPA is a rare form of vasculitis characterized by asthma, eosinophilia, and granulomatous or vasculitic involvement of several organs. The diagnosis and management of EGPA are often challenging and require an integrated, multidisciplinary approach [3]. In this case, the initial inappropriate use of inhaled corticosteroids failed to relieve the symptoms until more aggressive treatment with prednisolone was initiated. This was followed by 45 mg prednisolone treatment, with tapering of prednisone, without relapse over 3 months follow-up. In this case, despite unexplained manifestations, including intermittent remarkably increased eosinophilia, recurrent wheezing, and breathlessness, EGPA was completely ignored until the patient presented with aggravated petechia in the bilateral lower extremities that progressed to pulmonary embolism.

Thus, in this rare case of EGPA, the patient was misdiagnosed for more than two years and received inappropriate drugs, all of which could have been prevented. This case highlights the importance of careful differential diagnosis of pulmonary asthma. It can provide important clues for clinicians to consider EGPA and enable a prompt and accurate diagnosis of this rare disease, thereby avoiding disease progression and inappropriate treatment.

## References

[1] Kumar AM, Bae GH, Besen J, Kwong BY, Rieger KE (2019). Eosinophilic granulomatosis with polyangiitis: histopathological confirmation despite negative serology. Am J Med.

[2] Galant-Swafford J, Geng B, Leibel S (2020). Two pediatric cases of ANCA-negative eosinophilic granulomatosis with polyangiitis successfully treated with dupilumab. J Allergy Clin Immunol Pract.

[3] Emmi G, Bettiol A, Gelain E, et al. Evidence-Based Guideline for the diagnosis and management of eosinophilic granulomatosis with polyangiitis. Nature reviews Rheumatology*.* 2023. 10.1038/s41584-023-00958-w 37161084

